# Electrocardiogram in Friedreich's ataxia: A short‐term surrogate endpoint for treatment efficacy

**DOI:** 10.1111/anec.12813

**Published:** 2020-11-05

**Authors:** Sandra Mastroianno, Michele Germano, Angela Maggio, Raimondo Massaro, Domenico Rosario Potenza, Aldo Russo, Massimo Carella, Giuseppe Di Stolfo

**Affiliations:** ^1^ Cardiology Unit Fondazione IRCCS Casa Sollievo della Sofferenza San Giovanni Rotondo Italy; ^2^ Child Neuropsychiatry Unit Fondazione IRCCS Casa Sollievo della Sofferenza San Giovanni Rotondo Italy; ^3^ Paediatric Oncology Unit Fondazione IRCCS Casa Sollievo della Sofferenza San Giovanni Rotondo Italy; ^4^ Medical Genetic Unit Fondazione IRCCS Casa Sollievo della Sofferenza San Giovanni Rotondo Italy

**Keywords:** electrocardiogram, Friedreich's ataxia, treatment

## Abstract

Friedreich's ataxia is a rare degenerative neuromuscular disorder, caused by a homozygous GAA triplet repeat expansion in the frataxin (FXN) gene, with a broad clinical phenotype characterized by progressive gait and limb ataxia, dysarthria, and loss of lower limb reflexes; cardiac involvement is represented by hypertrophic cardiomyopathy, ventricular arrhythmias, and sudden cardiac deaths. Currently, no definite therapy is available, while many drugs are under investigation; for this reasons, we need markers of short‐ and long‐term treatment efficacy acting on different tissue for trial evaluation. We describe the case of a 21‐year‐old patient affected by Friedreich's ataxia on wheel‐chair, with initial cardiac involvement and electrocardiographic features characterized by thiamine treatment‐related negative T wave and QTc variations. We discuss plausible physiopathology and potential ECG role implications as an intermediate marker of treatment response in future clinical trials considering patients affected by Friedreich's ataxia.

## 1 INTRODUCTION

Friedreich's ataxia is a rare recessive autosomic degenerative neuromuscular disorder, caused by a homozygous GAA triplet repeat expansion in the frataxin (FXN) gene, leading to reduced expression of frataxin, an essential and highly conserved protein relevant in mitochondrial iron homeostasis, particularly the de novo biosynthesis of iron–sulfur cluster proteins and heme biosynthesis (Payne & Wagner, 2012). The disease yields a broad clinical phenotype characterized by progressive gait and limb ataxia, dysarthria, and loss of lower limb reflexes progressive (Cook & Giunti, 2017); cardiac involvement is represented by hypertrophic cardiomyopathy, ventricular arrhythmias, and sudden cardiac deaths (Hanson et al., 2019).

The histological cardiac features are represented by cardiomyocytes hypertrophy, diffuse fibrosis, and focal myocardial necrosis (Koeppen, 2011). Diagnostic phenotyping of different cardiac disease stage is represented by initial cardiac hypertrophy and a final evolution in a dilated cardiomyopathy with contractile dysfunction secondary to fibrosis (Hanson et al., 2019).

Friedreich's ataxia is characterized by mitochondrial dysfunction and increased concentration of reactive oxygen species (ROS), based on different pathophysiological hypothesis (Abeti et al., 2018). Direct consequence of reduced expression of frataxin is the impairment of mitochondrial iron metabolism particularly in the biosynthesis of iron–sulfur cluster, an essential component of Complex I–III enzymes in respiratory electronic transport chain; several therapeutic approaches are under investigation targeting ROS to possibly restore the impaired energy generation related to these pathways.

Ventricular repolarization, in particular QT dispersion, is strictly associated to electrical instability leading ventricular arrhythmias and sudden cardiac death, and is affected by ROS state (Sovari et al., 2013); at the same time, ROS reduction by antioxidant agents reverses QT prolongation and reduces arrhythmia occurrence (Dey et al., 2018).

Cardiac structural features evolve during time according to disease stage, as recently proposed, from initial isolated electrocardiographic modifications to hypertrophic cardiomyopathy and interstitial edema, with final myocardial fibrosis and dilated pattern (Mavrogeni et al., 2020; Peverill, 2016).

While treatment‐induced reversible structural cardiac features, quantified by echocardiography, cardiac MRI, and cardiac CT, need long time to be detectable, ventricular repolarization may be modified in a shorter time scale, indeed yielding important prognostic implication in term of arrhythmia and sudden cardiac death.

Currently, no definite therapy is available, whereas many drugs are under investigation; for this reasons, we need markers of short‐ and long‐term treatment efficacy for trial evaluation.

Thiamine represents promising role in Friedreich's ataxia treatment (Costantini et al., 2013; Costantini et al., 2016), although we need further larger clinical investigation to reach a standardize treatment.

In our report, we underline the role of electrocardiogram as a marker of thiamine treatment, relaying on significant T wave morphology and QT variations after treatment and abrupt withdrawal.

## 2 CLINICAL REPORT

We describe the case of 21‐year‐old woman affected by Friedreich's ataxia, at an advanced stage on intermittent wheel‐chair support. Previous genetic analysis demonstrated GAA triplet expansion between 600 and 700 times. During cardiologic follow‐up, she underwent periodic check‐up by electrocardiogram, Holter ECG, and echocardiogram. Although patient control and treatment were impaired by disease denial, she accepted thiamine treatment, as suggested by neurologists.

**FIGURE 1:**
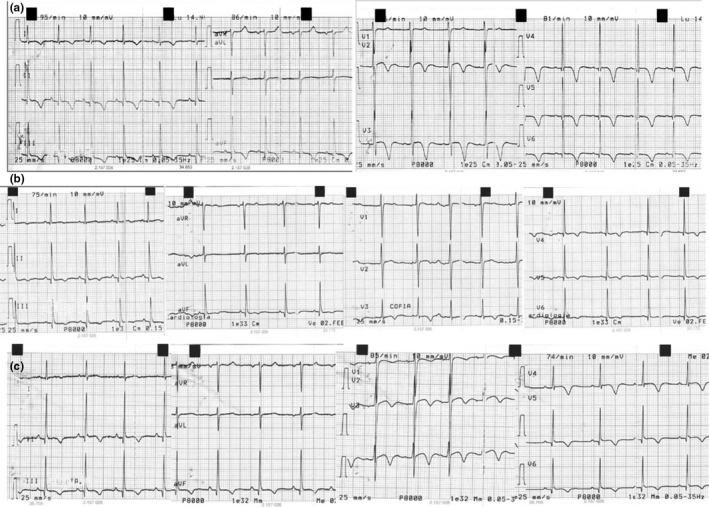
(a) Electrocardiogram before treatment; (b) Electrocardiogram during treatment; (c) Electrocardiogram after treatment withdrawal

Previous cardiological assessment showed mild symmetric left ventricular walls thickness, with both septum and posterior wall of 12 mm. ECG evaluation highlighted deep negative symmetric T wave in inferior leads and V2‐V6 (Figure 1a). Two years later, she underwent cardiac control after thiamine treatment start (1 gr three times a day). We noticed an initial reduction in left ventricular wall thickness to 10 mm; the most evident change was represented by impressive reduction of aforementioned negative T wave voltage, that turn to be asymmetric (Figure 1b). Unfortunately, one year later, the patient came back for cardiological assessment, after three months of treatment interruption. We observed deep symmetric negative T wave again, even if on less voltage amplitude (Figure 1c); on the other hands, left ventricular wall thickness remained 10 mm, on both septum and posterior wall. No difference emerged in terms of ventricular arrhythmia burden at several Holter ECG comparisons, only characterized by low frequency of single premature ventricular beats, fewer than one hundred; one more detail was represented by a lower rest heart rate during thiamine treatment (55 bpm) than before treatment (90 bpm) and after withdrawal (75 bpm). After strict comparison of electrocardiographic and echocardiographic parameters, together with treatment‐related T wave negative amplitude variations we observed QT prolongation normalized by thiamine. Although patient management was impaired by disease denial, we advice her to keep treatment according to neurologist suggestion, even if neurological evaluation by Friedreich's ataxia rating scale (FARS) was difficult to obtain; patient moods may affect FARS scoring, as raised by previous studies that have led to scale revision to a modified one (Rummey et al., 2019).

## 3 DISCUSSION

In our case, we noticed a clear relationship between QT prolongation and T wave morphology with thiamine treatment status. According to literature, Friedreich's ataxia is characterized by mitochondria dysfunction and ROS increase; thiamine treatment may rescue this pathological phenotype on both functional and structural level, underlined by electrocardiogram and echocardiography, respectively. ROS increase leads to myocardial edema, in part responsible for increased wall thickness at first echocardiographic control, while mitochondria imbalance alters ions flow across cellular membrane impairing cardiomyocytes repolarization and ST‐T phase on electrocardiogram. After two‐year treatment, echocardiography highlights wall thickness reduction, still evident after three months withdrawal. On the other hand, we noticed QT prolongation and deeper negative T wave before treatment and three months after withdrawal, with an evident reduction during thiamine consumption. Of course treatment results occur on a different time scale, as edema and cardiomyocytes hypertrophy need more time for a phenotype reversal and subsequent echocardiographic evidence than electrocardiographic modifications.

At same time, we noticed a slow heart rate reduction during treatment; although in Friedreich's ataxia only large myelinated fiber are involved, cardiac autonomic function, based on small and demyelinated fibers, is still under investigation. Nevertheless, there is evidence of increased heart rate at rest in affected patient, even without clear autonomic function impairment (Indelicato et al., 2018; Ingall & McLeod, 1991).

According to our literature revision, electrocardiogram may represent a valuable short‐term marker of cardiac disease reversal in patients with Friedreich's ataxia at early stage.

Ventricular repolarization abnormalities may represent an intermediate surrogate endpoint in clinical trials in patient ad early stage of cardiac involvement. Anyway, T wave morphology quantification could be difficult to realize, since a binary definition based on positive/negative morphology loses a great amount of information; we believe that a deeper ECG signal processing than standard time‐related interpretation, based on T wave area detection, although to be further investigated, could allow a better description of ventricular repolarization variations (Shang et al., 2019).

Since cardiomyopathy tends to be correlated with the clinical neurologic age of onset and the nucleotide triplet repeat length (i.e., markers of phenotypic disease severity) rather than the duration of disease or the severity of neurologic symptoms, different clinical items have to be addressed during treatment evaluation, as a reliable complete and effective patient treatment needs a combined therapy. For instance, the occurrence of improved FARS, meaning a better patient quality of life and a slow progression of neurological involvement, could not imply a mortality reduction secondary to cardiac threatening arrhythmias. For this reason, specific drug candidate may exert different effect on specific tissue on a diverse time scale based on disease stage; electrocardiogram may represent a good shot‐term surrogate endpoint for treatment evaluation.

## 4 CONCLUSION

In conclusion, we underline the prominent association between ventricular repolarization electrocardiographic features and thiamine treatment in a young patient affected by Friedreich's ataxia with initial cardiac involvement. Although previous scientific literature showed no evidence of strict correlations between ECG findings and disease progression on a long time scale, it would anyway imply a role in evaluation of cardiac metabolism regulation and reduction of ventricular repolarization dispersion depending on cardiac disease involvement. We suggest that ventricular repolarization electrocardiographic variations need to be deeply investigated in further studies as a plausible intermediate and objective marker of treatment response in clinical trials considering patients affected by Friedreich's ataxia at early stage.

## CONFLICT OF INTERESTS

Authors do not have conflict of interest.

## AUTHOR CONTRIBUTION

**Sandra Mastroianno**: Conceptualization—original draft—review and editing (equal). **Michele Germano**: writing—review and editing (equal). **Angela Maggio**: writing—review and editing (equal). **Raimondo Massaro**: writing—review and editing (equal). **Domenico Rosario Potenza**: review and editing (equal). **Aldo Russo**: review and editing (equal). **Massimo Carella**: review and editing (supporting). **Giuseppe Di Stolfo**: Conceptualization; Writing—original draft (supporting); Writing—review and editing (equal).

## ETHICAL APPROVAL

This case report was writing respecting patient confidentiality and privacy. Patient had the opportunity to read the present case report and had no objections to the final abstract.
